# Allelic diversity and selection at the MHC class I and class II in a bottlenecked bird of prey, the White-tailed Eagle

**DOI:** 10.1186/s12862-018-1338-3

**Published:** 2019-01-05

**Authors:** Piotr Minias, Ewa Pikus, Dariusz Anderwald

**Affiliations:** 1 Department of Biodiversity Studies and Bioeducation, Faculty of Biology and Environmental Protection, University of Łódź, Banacha 1/3, 90-237 Łódź, Poland; 2 Eagle Conservation Committee, Niepodległości 53/55, 10-044 Olsztyn, Poland

**Keywords:** Diversifying selection, *Haliaeetus albicilla*, Major histocompatibility complex, Population bottleneck, Trans-species polymorphism, White-tailed eagle

## Abstract

**Background:**

Genes of the Major Histocompatibility Complex (MHC) are essential for adaptive immune response in vertebrates, as they encode receptors that recognize peptides derived from the processing of intracellular (MHC class I) and extracellular (MHC class II) pathogens. High MHC diversity in natural populations is primarily generated and maintained by pathogen-mediated diversifying and balancing selection. It is, however, debated whether selection at the MHC can counterbalance the effects of drift in bottlenecked populations. The aim of this study was to assess allelic diversity of MHC genes in a recently bottlenecked bird of prey, the White-tailed Eagle *Haliaeetus albicilla*, as well as to compare mechanisms that shaped the evolution of MHC class I and class II in this species.

**Results:**

We showed that significant levels of MHC diversity were retained in the core Central European (Polish) population of White-tailed Eagles. Ten MHC class I and 17 MHC class II alleles were recovered in total and individual birds showed high average MHC diversity (3.80 and 6.48 MHC class I and class II alleles per individual, respectively). Distribution of alleles within individuals provided evidence for the presence of at least three class I and five class II loci the White-tailed Eagle, which suggests recent duplication events. MHC class II showed greater sequence polymorphism than MHC class I and there was much stronger signature of diversifying selection acting on MHC class II than class I. Phylogenetic analysis provided evidence for trans-species similarity of class II, but not class I, sequences, which is likely consistent with stronger balancing selection at MHC class II.

**Conclusions:**

Relatively high MHC diversity retained in the White-tailed Eagles from northern Poland reinforces high conservation value of local eagle populations. At the same time, our study is the first to demonstrate contrasting patterns of allelic diversity and selection at MHC class I and class II in an accipitrid species, supporting the hypothesis that different mechanisms can shape evolutionary trajectories of MHC class I and class II genes.

**Electronic supplementary material:**

The online version of this article (10.1186/s12862-018-1338-3) contains supplementary material, which is available to authorized users.

## Background

The Major Histocompatibility Complex (MHC) multigene family constitutes an essential part of vertebrate immune system, as MHC receptors bind and recognize peptides derived from pathogen processing [1]. Each MHC molecule has a peptide-binding groove which binds to a limited range of foreign peptides and, thus, the number of different MHC molecules expressed within an organism determines the spectrum of pathogens that can be recognized and killed via adaptive immune defences. Within-individual diversity of MHC alleles is primarily limited by the number of duplicated MHC loci [2]. In birds, the number of MHC loci is relatively low in ancestral lineages, but extensive MHC duplication seems to have occurred in more recently evolved taxa [3]. A single dominantly expressed MHC gene at both class I and class II has been recorded in some old non-passerine lineages such as galliforms or penguins (so called ‘minimal essential‘ MHC [4]), whereas tens of putatively transcribed loci have been described for some passerine species [5–7]. The apparent fitness advantage of MHC heterozygosity at the individual level is often reflected by an astonishing allelic diversity maintained in natural populations of vertebrates. For example, over 3500 MHC class I alleles were found within a Central European population of sedge warblers *Acrocephalus schoenobaenus* [7] and nearly a thousand MHC class II alleles were recorded in the common yellowthroat *Geothlypis trichas* [5]. This extreme MHC polymorphism is thought to be generated and maintained via pathogen-mediated diversifying and balancing selection [8], acting through the mechanisms of overdominance [9], negative frequency-dependence [10], and fluctuating selection [11]. Strong balancing selection can maintain alleles for long evolutionary periods, often beyond neutral expectations. Persistence of adaptive MHC alleles beyond species diversification due to balancing selection produces a pattern where some alleles are more similar between species than within species, which is referred to as trans-species polymorphism [12].

There are two major types of transmembrane MHC receptors: class I and class II. Class I receptors are expressed on nearly all nucleated vertebrate cells and are responsible for the presentation of antigens from within a cell to cytotoxic T-cells [1]. In contrast, class II receptors are expressed on specific antigen-presenting cells, such as dendrintic cells, monocytes or B cells, which phagocytose extracellular antigens and present them to T helper cells [1]. Since MHC class I and class II initiate adaptive immune response against intra- and extracellular pathogens, respectively (but see [13] for antigen cross-presentation mechanisms), there might be differences in the strength of pathogen-mediated selection acting on these genes, resulting in different evolutionary trajectories and contrasting patterns of polymorphism between class I and class II genes. In birds, the relative exposure rate to intra- and extracellular pathogens can vary with morphology, habitat, general ecology, and life-history strategies [14], and the same set of traits is expected to govern selection patterns at the MHC. For example, it has been shown that migratory and colonial avian species show stronger signature of diversifying selection acting on MHC class II genes, likely because of elevated transmission rates of pathogens in socially breeding species and exposure to more diverse fauna of pathogens during migration [15]. Selection patterns at the MHC can also differ between phylogenetic lineages or taxa that show strong ecological divergence. A recent analysis of selection across the avian tree of life provided evidence for striking differences in the strength of diversifying selection acting on MHC class I and class II in passerine and non-passerine birds [16]. Specifically, non-passerines showed stronger selection at MHC class II (presentation of extracellular antigens), while passerines had stronger signature of selection at MHC class I (presentation of intracellular antigens) [16]. It was concluded that non-passerine birds are primarily selected to recognize a broader spectrum of extracellular versus intracellular pathogens, which could be attributed to their larger body sizes (body size is thought to determine extracellular pathogen richness [17]) and stronger preferences for freshwater aquatic habitats (aquatic environments are thought to accommodate more diverse faunas of extracellular, but not necessarily intracellular pathogens [18, 19]). In spite of these general findings, most molecular research on MHC in non-model avian taxa focus on a single MHC class, while signatures of pathogen-mediated selection have rarely been compared between MHC class I and class II within a single population or species [20–22].

A robust methodological pipeline to genotype MHC class I and class II in diurnal birds of prey from three distinct orders of Accipitriformes, Cathartiformes and Falconiformes has been developed as a part of pioneer research by Alcaide et al. [23, 24]. Since then, an extensive scientific effort has been devoted to examine the patterns of MHC polymorphism and its consequences in natural populations of several falcon species [25–28]. Thus, it may seem surprising that only few accipitrids had their MHC characterized in detail up to date [29–31], especially considering that the order of Accipitriformes includes most of diurnal raptors, such as hawks, eagles, and Old World vultures. Here, we used Next-Generation Sequencing methods (Illumina MiSeq) to genotype MHC class I and class II in a recently bottlenecked accipitrid species, the White-tailed Eagle *Haliaeetus albicilla*.

The genus of *Haliaeetus* contains eight species of large diurnal raptors, often referred to as sea eagles, that have wide distribution around the globe [32]. They are widely considered as flagship species for conservation and often serve as umbrella species to protect animal communities or entire ecosystems. White-tailed Eagle has a large breeding range spanning from Central and Northern Europe (including Iceland and SW costs of Greenland) through the Middle East and Central Siberia to the Pacific coast of Asia [32]. A rough estimate of the global population size is 25–50 thousand mature individuals, including 9–12 thousand pairs breeding in Europe [33]. Nowadays, Norway and Russia are the major strongholds of the species in Europe, as they hold over half of the European population [33]. The White-tailed Eagle was formerly a common species across Europe, but in nineteenth century the population started to decline following rapid landscape changes (intensification of agriculture) and direct persecution. The species went extinct in the Western Europe (including Great Britain) by the early 1900s. High environmental pollution resulting in low reproductive success led to further extinctions of many local Central European populations (e.g. in Austria, Czech Republic, and Slovakia) in the middle of twentieth century. By the 1970s, central and northern European population reached the most severe demographic bottleneck, reaching approximately one thousand of pairs, 700–800 of which bred at the Atlantic coast of Norway [34]. With the prohibition of chemical pesticides (DDT and PCBs) in 1970s and strict legal protection of the species, population started to recover and many previously abandoned areas become recolonized, occasionally helped with reintroduction programs [35]. In Poland, the population size of White-tailed Eagles was estimated at 80–90 breeding pairs during 1970s and it has recently increased to 1100–1200 pairs, as estimated in 2017 (Eagle Conservation Committee, unpublished data). Despite this severe population bottleneck, the species seems to have retained relatively high genetic variation, as assessed with neutral genetic markers [34, 36]. However, as far as we aware, polymorphism of innate or adaptive immune genes has never been examined in this species. The main goals of this study were to: *i*) assess diversity of MHC genes within a recovered Central European population of the White-tailed Eagle; *ii*) examine historical patterns of diversifying selection acting on these genes; and *iii*) compare evolutionary mechanisms responsible for maintaining polymorphism at MHC class I and class II in this species.

## Methods

### Sample collection

Samples for this study were collected in 2017 from the core population of White-tailed Eagles in the northern Poland. In total, 67 White-tailed Eagle nestlings from 44 broods (1.52 ± 0.08 [SE] nestling per brood) were sampled. Between one and three growing (in shafts) feathers lost or broken during standard ringing procedures were collected from each bird into 96% ethanol. All samples were collected between 13 May and 03 June. All experiments complied with the current laws of Poland (Act on Nature Conservation from 16 April 2004, Journal of Laws from 2004, No. 92, item 880) and were approved by the Local Bioethical Commission for Animal Welfare in Łódź and the General Environmental Protection Directorate in Poland.

### Amplification and Illumina sequencing

Nuclear DNA was extracted from feathers using Bio-Trace DNA Purification Kit (EURx, Gdańsk, Poland). For this purpose, the tip of feather shaft filled with blood was cut with a sterile blade and DNA isolation followed the manufacturer’s protocol. The methods yielded an average DNA concentration of 189.0 ± 9.5 [SE] ng/μl. To genotype MHC in the White-tailed Eagle we used degenerate primers previously developed for other accipitrid species: *MHCI-int2F* and *MHCI-ex4R* for class I [24] and *Acc2FC* and *Acc2RC* [23] for class II. The first pair of primers amplifies MHC class I exon 3 by binding to the flanking region of intron 2 and conserved region of exon 4, while the second pair of primers amplifies MHC class II exon 2 by binding to the flanking regions of introns 1 and 3. Previously, both pairs of primers were successfully tested for MHC cross-amplification in a large spectrum of accipitrid species from different genera [23, 24]. We genotyped MHC class I exon 3 and MHC class II exon 2 because they form a peptide-binding groove of MHC molecules and most MHC research in non-model avian species has focused exclusively on these regions [16]. For example, polymorphism of class I exon 2 was examined only for a handful of species, mostly domestic ones [e.g. 37, 38], and consequently almost no information on the allelic diversity and selection signature at this exonic region exist for wild birds, including accipitrids.

PCR amplifications were conducted in a final volume of 20 μl containing 10 μl of 2X HotStarTaq Plus Master Mix Kit (Qiagen, Venlo, Netherlands), 50–150 ng of genomic DNA and 0.2 μM of each primer. Amplifications of MHC followed the steps of: (i) initial denaturation at 94 °C for 5 min; (ii) 25 (class I) or 30 (class II) cycles of denaturation at 94 °C for 40 s followed by 40 s at 56 °C annealing temperature and elongation at 72 °C for 40 s; (iii) final elongation at 72 °C for 10 min. Relatively low number of amplification cycles (25–30) was used to minimize the risk of chimera formation. All PCR products were evaluated by electrophoresis on 2% agarose gel to detect positive amplifications. Amplifications were completed using fusion primers containing Illumina adapter sequences, a 7-bp barcode that indicated sample identity, and a pair of either the MHC class I or II primers. Fourteen samples were amplified in two independent PCRs to obtain technical replicates. All PCR products were purified and their concentrations were determined with Quant-iT PicoGreen dsDNA Assay Kit (Thermo Fisher Scientific, Waltham, MA, USA). Equimolar quantities of PCR products were pooled to form a library using NEBNext DNA Library Prep Master Mix Set for Illumina (New England Biolabs, Ipswich, MA, USA). The library was sequenced using Illumina v2 Kit at a 2 × 250 bp paired-end Illumina MiSeq platform.

### Processing of Illumina data and MHC allele validation

Processing of raw Illumina MiSeq data was conducted using Amplicon Sequencing Analysis Tools (AmlpiSAT) web server developed by Sebastian et al. [39] and followed the algorithm recommended by Biedrzycka et al. [7]. First, pair-ended reads were merged with AmpliMERGE tool that is based on FLASH algorithm with optimum overlapping parameters [40]. Then, AmpliCHECK tool with default settings (minimum per amplicon frequency of 1%) was used for the preliminary exploration of the data set. There was a clear predominance (88.8%, *n* = 711) of 412-bp sequences at the MHC class I. Twelve sequences of similar lengths (± 2 bp) were retrieved, but they showed minor frequencies (1.11–2.04%) and each variant was recorded in a single individual. Thus, we considered these sequences as originating from genotyping errors. Also, 67 short-length (67–298 bp) sequences were considered as genotyping artefacts. Only 269-bp sequences were retrieved at the MHC class II. Based on AmpliCHECK results, the exact read length of 412 bp and 269 bp for MHC class I and class II, respectively, was set in further data processing. AmpliSAS tool was used for the final de-multiplexing, clustering, and filtering of Illumina reads. Default parameters for Illumina data were used for clustering (1% substitution errors, 0.001% indel errors, and 25% minimum dominant frequency). In the filtering step, we discarded chimeras and sequences that had less than 3% frequency, as well as those that were recorded in a single sample. Minimum amplicon depth was set to 100 reads, while maximum amplicon depth was set to 5000 reads because of AmpliSAS performance reasons. After processing, the average number of reads per sample was 2402 ± 164 [SE]. Reproducibility of alleles between technical replicates (amplicon depth > 1000) was 90.1%, which is consistent with an expected repeatability of MHC allele calling for the AmpliSAS software [41]. MHC class I and class II alleles were aligned separately in Geneious v10.0.5 (Biomatters Ltd., Auckland, New Zealand). Intron regions were removed from the alignments, retaining a 273-bp fragment of MHC class I exon 3 (total exon length: 276 bp) and 258-bp fragment of MHC class II exon 2 (total exon length: 270 bp).

### Sequence polymorphism and selection

**Fig. 1:**
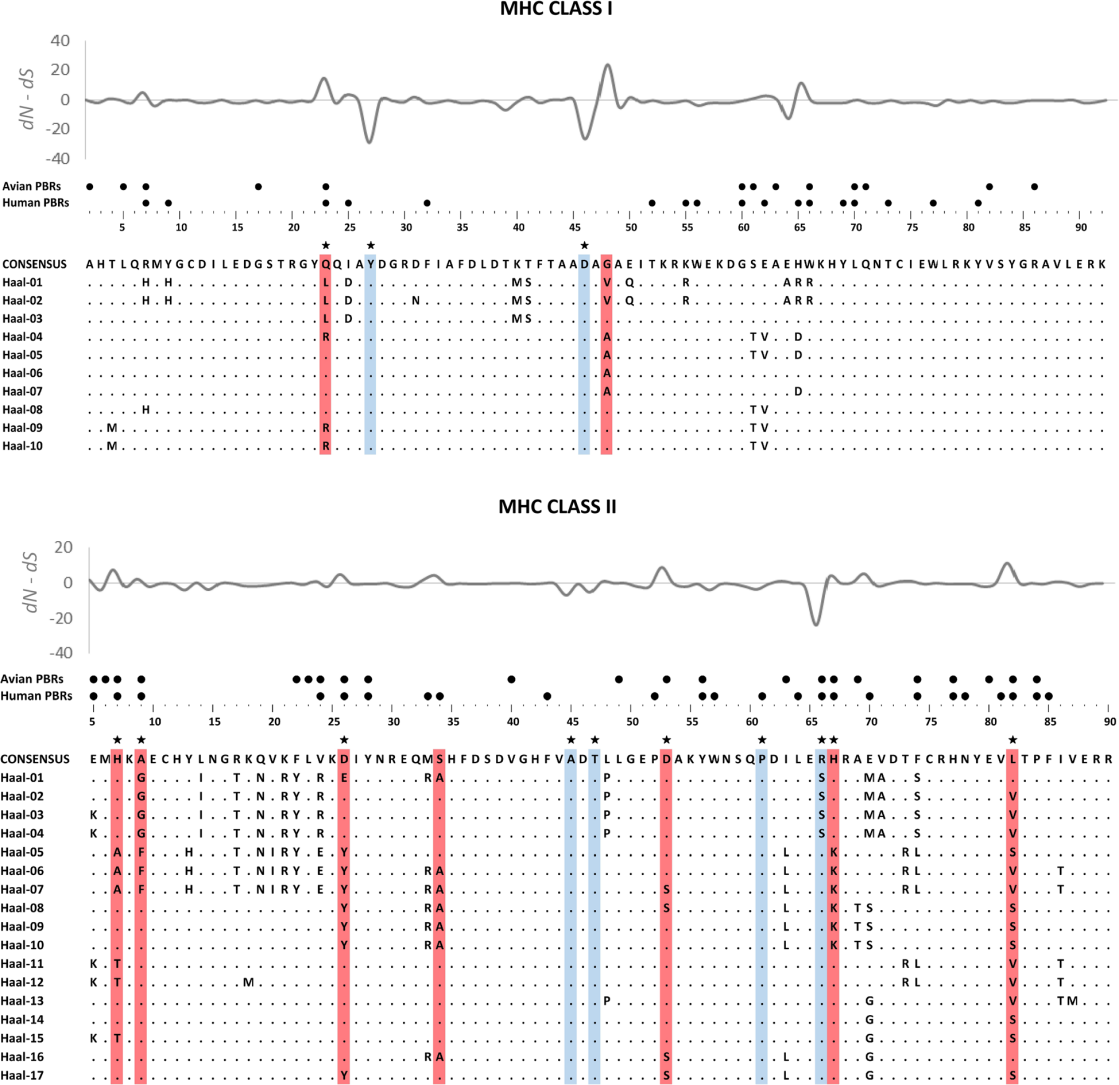
Alignments of amino acid sequences of MHC class I exon 3 and MHC class II exon 2 in the White-tailed Eagle. Dots in the alignments indicate the same amino acid as the top consensus sequence. Amino acid residues under positive selection are marked with red, under negative selection are marked with blue. Positively and negatively selected residues inferred for non-recombinant fragments (sequence partitions identified with GARD analysis) are marked with stars. Large dots (●) above the alignment indicate peptide-binding residues (PBRs) of humans (according to Saper et al. [46] for class I and Brown et al. [47] for class II) and putative PBRs of non-passerine birds (according to Minias et al. [14]). Spatial variation in the selection parameter (*dN – dS*) is shown at the top of each alignment

Sequence polymorphism was assessed as the number of polymorphic sites, total number of mutations, average nucleotide diversity, and average number of nucleotide differences using DnaSP v.6.10.3 [42]. To infer selection acting on MHC class I exon 3 and MHC class II exon 2 we inferred relative rates of nonsynonymous (amino acid altering) and synonymous (silent) nucleotide substitutions. Under positive (diversifying) selection, nonsynonymous substitutions are expected to accumulate faster than synonymous substitutions (new allelic variants are promoted), while the opposite pattern occurs under negative (purifying) selection, which purges most allelic variants that arise through point mutations. Thus, the relative rate of nonsynonymous substitutions per nonsynonymous site (*dN*) to synonymous substitutions per synonymous site (*dS*) is commonly used a measure of selection acting on the sequence over evolutionary time, where *dN*/dS < 1 indicates negative selection, *dN*/*dS* > 1 indicates positive selection, and dN/dS ≈ 1 indicates neutral evolution [43]. We assessed codon-specific signatures of positive (pervasive diversifying) and negative (pervasive purifying) selection using two different (Bayesian and maximum-likelihood) approaches implemented in HyPhy software [44], which was run through the Datamonkey Adaptive Evolution Server [45, 46]. First, we used Fast Unconstrained Bayesian AppRoximation (FUBAR) method, which uses a Markov Chain Monte Carlo (MCMC) routine that ensures robustness against model misspecifications and leaves the distribution of selection parameters essentially unconstrained [47]. Second, we used Fixed Effects Likelihood (FEL) method, which shows intermediate conservatisms in term of type I error when compared with other maximum-likelihood approaches implemented in Hyphy [48]. We also used Mixed Effect Model of Evolution (MEME) [49] to identify sites under episodic diversifying selection, i.e. evolving under positive selection at a proportion of branches. All analyses were run with default settings and input trees built from the sequence alignments. Amino acid residues with > 0.95 Bayesian posterior probabilities (FUBAR) or *p* < 0.05 (FEL and MEME) were considered to have enough support for positive or negative selection. We also used Genetic Algorithm for Recombination Detection (GARD) implemented in HyPhy to search for evidence of recombination breakpoints and identify putative recombinant fragments (partitions) in MHC class I and class II sequences [50]. Significance threshold of *p* = 0.1 was used to identify recombination breakpoints. Since no unique tree topology can describe the evolutionary history of recombinant sequences, selection inference on recombinant data may produce false positives [51]. Thus, we re-run all codon-specific selection models using a unique phylogenetic history for each detected recombination block, as identified with GARD. The *dN*/*dS* ratio was measured for three different subsets of amino acid residues: *i*) all residues, (*ii*) human peptide-binding residues (PBRs), as identified based on the crystallography models of human MHC molecules [52, 53], and *iii*) putative PBRs of non-passerine birds, as identified based on the global analysis of selection at the avian MHC [16] (Fig. 1).

### Phylogenetic clustering

To assess phylogenetic relationships between MHC sequences of the White-tailed Eagle and other accipitrid species, we used BLAST search (as implemented in Geneious v10.0.5) for the two query White-tailed Eagle alleles that showed lowest pairwise identity within each MHC class. In each search we retrieved eight sequences most similar to the query sequence (no more than two sequences per species). Phylogenetic relationships were inferred using approximately-maximum-likelihood approach, as implemented in FastTree v2.1.5 [54]. FastTree software constructs an initial tree with neighbour-joining and then refines its topology with subtree-pruning-regrafting (SPRs), minimum-evolution nearest-neighbour interchanges (NNIs), and maximum-likelihood NNIs. Tree topologies produced with this approach show similar accuracy to topologies produced with standard maximum likelihood methods [55]. General time-reversible (GTR) model of nucleotide substitution with a discrete Gamma distribution was used to account for different rates of evolution at different sites and for uncertainty in these rates [56]. Local support values were computed based on the Shimodaira-Hasegawa test [57]. Sequences from the domestic chicken *Gallus gallus* (GenBank accession nos.: AM419160 for class I, AM489767 for class II) were used as outgroups.

## Results

**Fig. 2:**
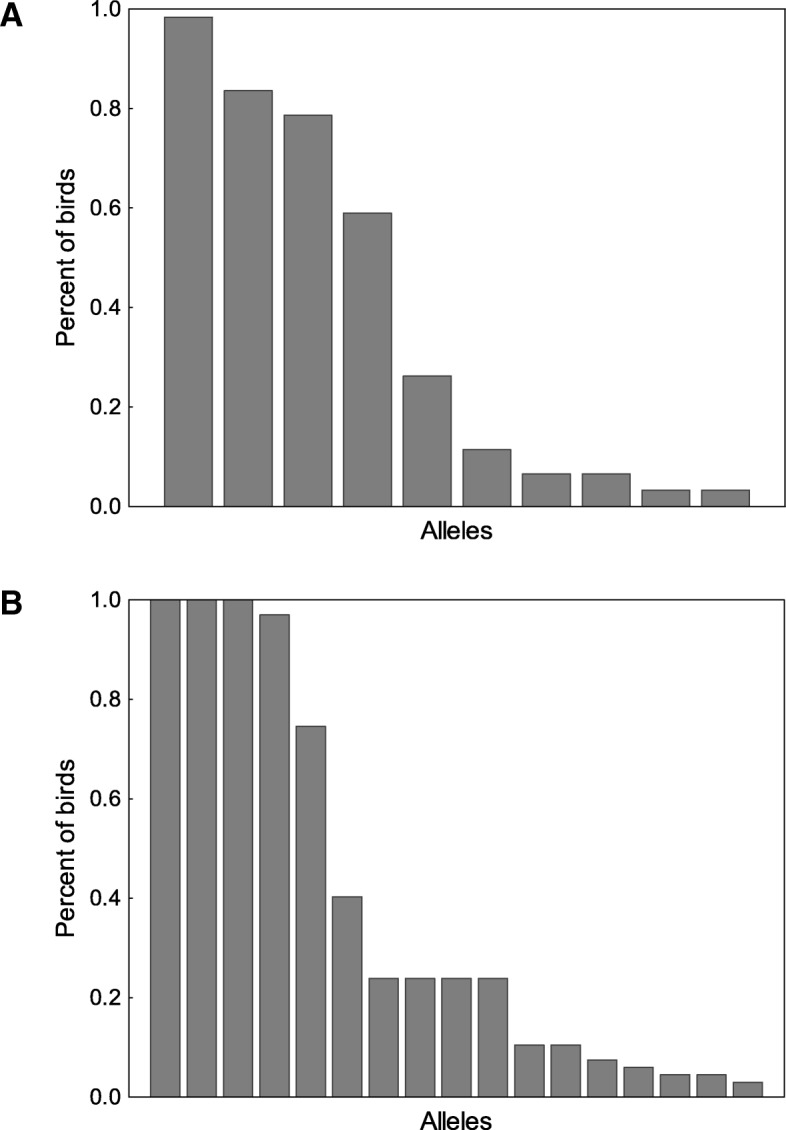
Percent of White-tailed Eagle individuals where particular MHC class I (**a**) and MHC class II (**b**) alleles were found

We found that White-tailed Eagle nestlings had lower allelic diversity at MHC class I than class II. After clustering and filtering of Illumina reads, 10 MHC class I and 17 MHC class II alleles were retained (Fig. 1). These alleles corresponded to 9 and 15 unique amino acid sequences, respectively, all of which were putatively functional. One MHC class I allele was found in > 90% of individuals, whereas three MHC class II alleles were found in all genotyped birds (Fig. 2). The mean number of alleles per individual was 3.80 ± 0.20 and 6.48 ± 0.20 for MHC class I and class II, respectively. A maximum number of MHC class I and class II alleles per individual was six (*n* = 7 individuals) and nine (*n* = 11 individuals), respectively, which indicated for the presence of at least three class I and five class II loci in the White-tailed Eagle. MHC class II sequences had higher proportion of polymorphic sites (50 polymorphic sites per 258 bp) and higher total number of mutations (η = 60) than MHC class I sequences (27 polymorphic sites per 273 bp and η = 29 mutations). The average nucleotide diversity was 0.043 ± 0.010 [SD] for MHC class I and 0.087 ± 0.007 [SD] for MHC class II. Consistently, the average number of nucleotide differences was 11.9 and 22.4 for MHC class I and class II, respectively.

**Fig. 3:**
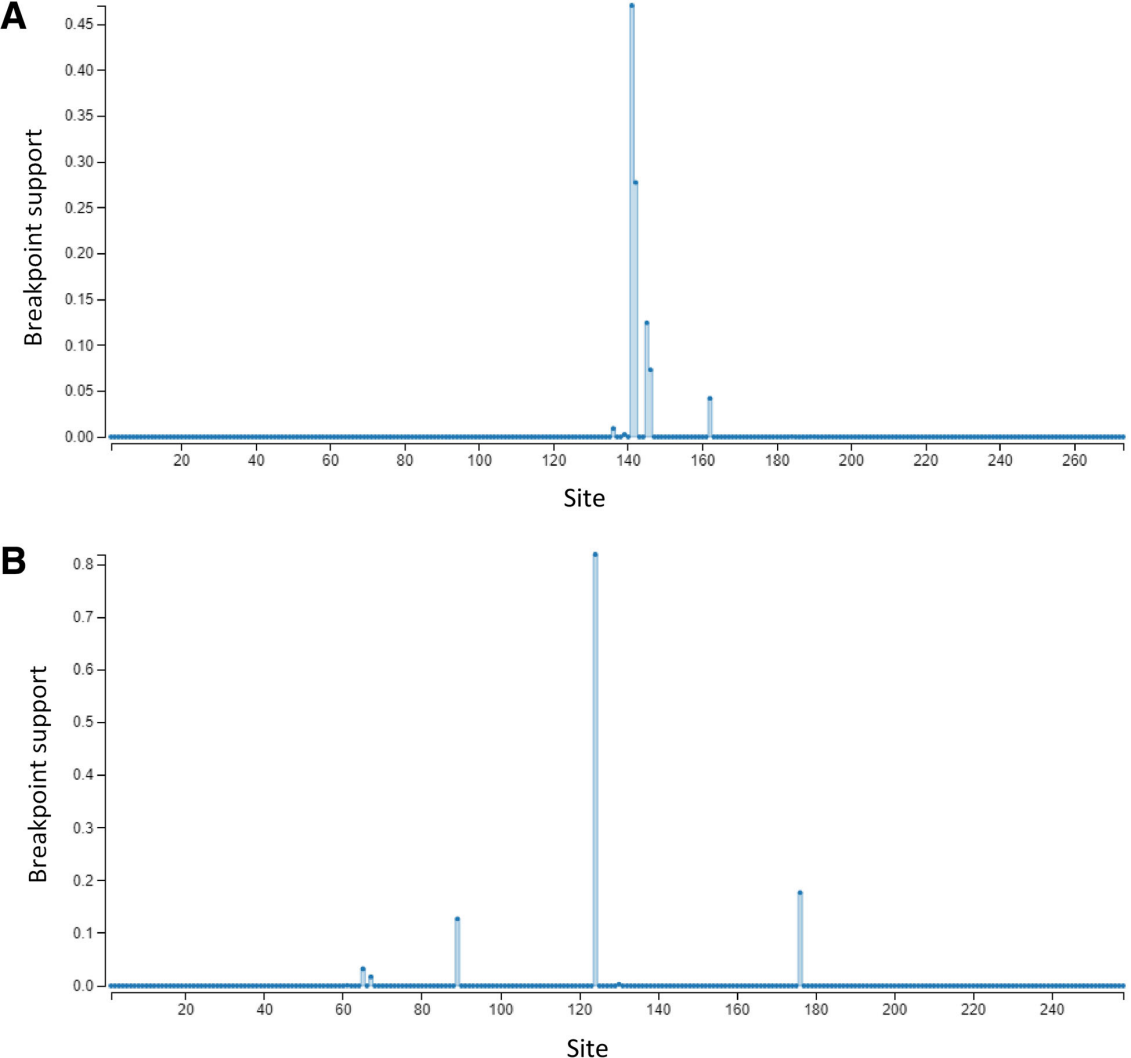
Model-averaged support for recombination breakpoints (as assessed with GARD method) and their location along the MHC class I (**a**) and class II (**b**) sequences of the White-tailed Eagle

We found evidence for much stronger positive (diversifying) selection acting on MHC class II than class I in the White-tailed Eagle. Bayesian methods (FUBAR) identified seven sites under positive selection within MHC class II exon 2, while only two sites were recognized as under positive selection within MHC class I exon 3 (Fig. 1). Similarly, maximum-likelihood approach (FEL) recognized four sites under positive selection at MHC class II, whereas none of the sites were recognized as positively selected at MHC class I. Finally, we found evidence for episodic diversifying selection (as assessed with MEME) acting on five MHC class II sites and only one MHC class I site. All three measures of *dN*/*dS* ratio (calculated for the entire sequence or exclusively for codons identified as putative PBRs in birds or humans) were higher at MHC class II than class I (Table 1). The greatest difference was observed at the putative avian PBRs (Fig. 1), where strong diversifying selection was observed for MHC class II (*dN*/*dS* = 3.64), while signature of diversifying selection at MHC class I was very weak (*dN*/*dS* = 1.11). GARD analysis identified a single recombination breakpoint in both class I (at 140 bp) and class II (at 123 bp) alignments (Fig. 3). Re-running codon-specific selection models for non-recombinant fragments of sequences did not qualitatively change the results and provided further support for stronger positive (diversifying) selection at MHC class II than class I, as assessed with the number of positively selected sites and *dN*/*dS* ratios (Table 1).

**Table 1:** Signatures of selection at MHC class I exon 3 and MHC class II exon 2 of the White-tailed Eagle, as measured with the number of residues under positive (pervasive diversifying) and negative (purifying) selection (assessed with FUBAR and FEL methods) and the relative rates of nonsynonymous to synonymous substitutions (*dN/dS*) at: *i*) all residues; *ii*) human peptide-binding residues (PBRs); and *iii*) putative PBRs of non-passerine birds

MHC class	Exon	Seq. length	No. of alleles	*dN/dS*			Method	No. of residues	
				All residues	Human PBRs	Avian PBRs		Positive selection	Negative selection
Class I	3	273 bp	10	0.82 (0.78)	1.71 (1.75)	1.11 (1.07)	FUBAR	2 (1)	2 (1)
							FEL	0 (0)	2 (2)
Class II	2	258 bp	17	1.47 (1.30)	2.93 (2.53)	3.64 (3.31)	FUBAR	7 (6)	4 (4)
							FEL	4 (3)	6 (5)

Selection estimates for non-recombinant fragments (sequence partitions identified with GARD analysis) are shown in brackets. Human PBRs were classified according to the crystallographic structure of MHC molecules (class I: Saper et al. [52]; class II: Brown et al. [53]), while putative avian PBRs were classified according to the global analysis of selection at the MHC of birds [16]

**Fig. 4:**
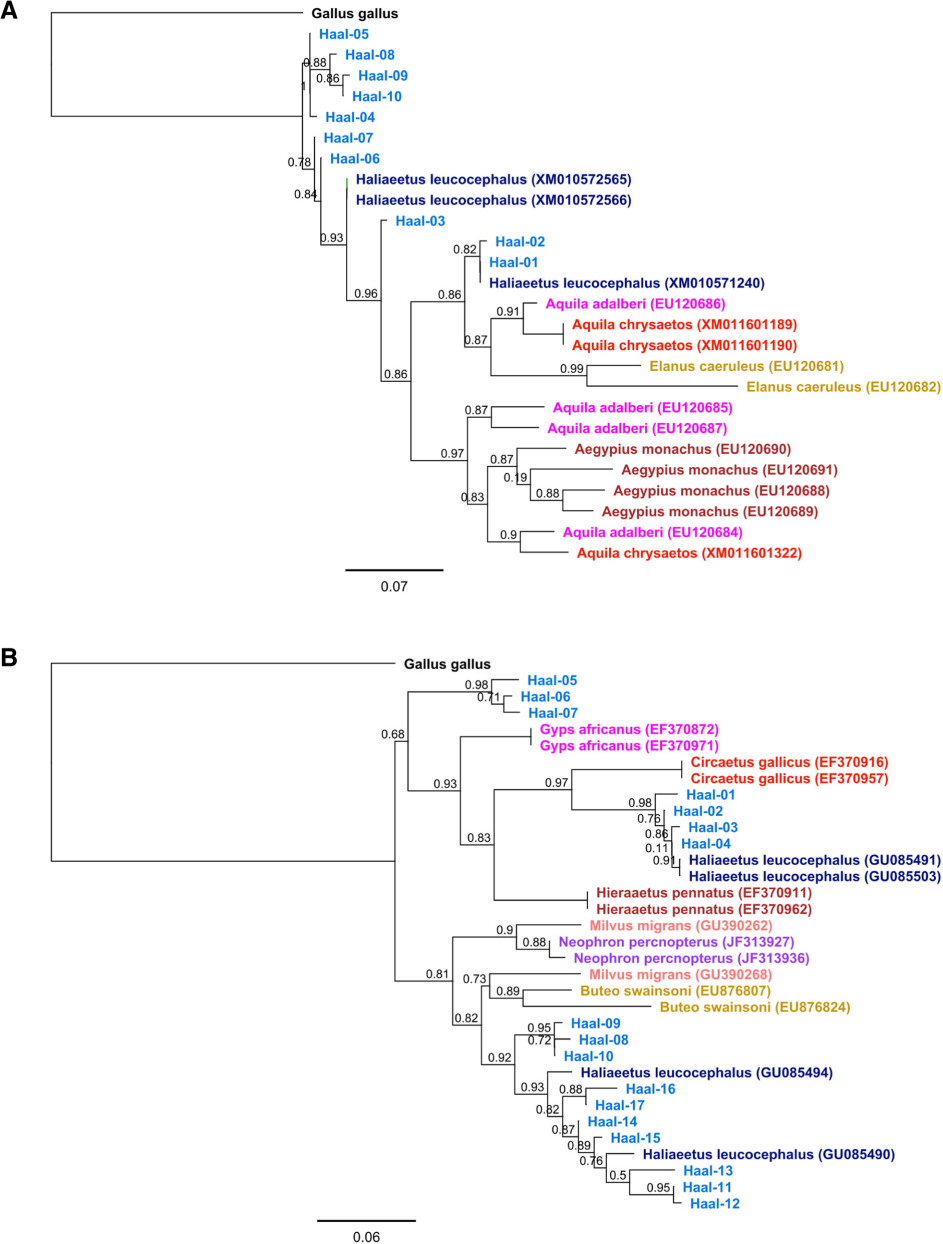
Consensus maximum likelihood topology for MHC class I exon 3 (**a**) and MHC class II exon 2 (**b**) in the White-tailed Eagle and other Accipitridae species. Local bootstrap support is provided at each node. Scale bar indicates genetic distance in units of nucleotide substitutions per site. Domestic chicken was used as an outgroup

Both MHC class I and class II sequences of the White-tailed Eagle showed high pairwise identity with sequences of a congeneric species, the Bald Eagle (90.8–100% pairwise identity for MHC class I; 85.3–99.6% pairwise identity for MHC class II). All MHC class I sequences of both *Haliaeetus* species clustered closely in a phylogenetic analysis and showed little evidence of trans-species polymorphism with sequences of accipitrids from other genera (Fig. 4). In contrast, MHC class II sequences of *Haliaeetus* formed three distinct clusters that were separated with sequences of other accipitrids, producing a pattern of trans-specific similarity (Fig. 4).

## Discussion

In this study we used Illumina MiSeq sequencing to genotype MHC class I and class II in White-tailed Eagle nestlings from the core Central European (Polish) population. Despite recent demographic bottleneck, we found moderate level of MHC diversity in our sample and found evidence for the presence of at least three class I and five class II loci in the White-tailed Eagle, as estimated with the maximum number of MHC alleles recorded per individual. MHC class II showed higher allelic diversity than MHC class I and there was much stronger signature of diversifying selection acting on MHC class II than class I. Finally, phylogenetic analysis of MHC sequences in White-tailed Eagle and other accipitrid species provided support for trans-specific similarity of class II, but not class I sequences.

Despite a severe demographic decline in the second half of twentieth century, European populations of the White-tailed Eagle have apparently retained significant level of genetic diversity. Analysis of neutral (both nuclear and mitochondrial) genetic markers in the extant Central European populations revealed no signs of recent genetic bottleneck and indicated that sustained genetic diversity had a predominantly local origin with little contribution of effective dispersal [34–36]. It has been argued that long generation time of the White-tailed Eagle (average and maximum lifespan in the wild was estimated at 17 and 26 years, respectively [32]) might have minimized genetic drift and acted as an intrinsic buffer against rapid loss of genetic diversity, especially in the case of relatively short bottleneck episodes [34]. This scenario was consistent with theoretical simulations showing that < 5% of the original heterozygosity is expected to be lost during a 30-year bottleneck which reduced the effective population size of White-tailed Eagles from 300 to 30 reproducing pairs [34]. Our results on MHC allelic diversity seem to support these conclusions, as we found moderately high variation at both MHC class I and class II in White-tailed Eagles from northern Poland. Although the total number of MHC amino acid variants recorded in our study population was moderate (9 MHC class I and 15 MHC class II variants recorded in 67 nestling from 44 families), individuals showed high average MHC diversity (3.80 and 6.48 MHC class I and class II alleles per individual, respectively). The level of MHC polymorphism in the White-tailed Eagle, as revealed in our study, was comparable to other accipitrid species that have broad distribution ranges and large population sizes. For example, 20 MHC class II alleles were isolated from Swainson’s Hawks *Buteo swainsoni* (*n* = 20) wintering in Argentina [31], while 13 MHC class II alleles were detected in eight Black Kites *Milvus migrans* from Spain [29]. Seventeen MHC class II alleles were identified within continental (Iberian) population of the Egyptian Vulture *Neophron percnopterus* (*n* = 96 individuals), while ten and nine alleles were found in the islandic populations from Canaries (*n* = 236 individuals) and Baleares (*n* = 36 individuals), respectively [30]. Only three MHC class II alleles were recovered from 32 individuals of an island endemic species, the Galápagos Hawk *Buteo galapagoensis* [31], indicating that viable accipitrid populations can persist with extremely low MHC variation. We are aware of no previous research on MHC class I diversity in accipitrid birds of prey.

Our study provided support for the presence of at least three class I and five class II loci in the White-tailed Eagle, which is unusual when compared with other birds of prey. In general, non-passerine birds have low number of MHC loci, which is thought to be an ancestral state in avian evolution [3]. Two MHC class II loci were recorded in most accipitrid species studied in detail up to date [29–31], while the presence of three loci was suggested for the Red Kite *Milvus milvus* [58]. Four MHC class II loci were found in non-passerines from several other taxonomic orders, e.g. in Anseriformes, Pelecaniformes, or Procellariiformes [58–60]. So far, Blakiston’s Eagle-owl *Bubo blakistoni* yielded the highest estimate of MHC class II loci among non-passerines, as up to 16 alleles were detected per individual, indicating for the presence of eight class II loci in this species [61]. Surprisingly, much fewer (1–3) MHC class II loci have been reported for other owl species, although these estimates were based on considerably smaller sample sizes [23, 62]. Similar pattern has been found in Galliformes, as most phasianid species tend to have 2–3 MHC class II loci [22, 63–66], while seven loci were identified in the Japanese Quail *Coturnix japonica* [67]. This suggests that, as in the case of White-tailed Eagle and other accipitrids, closely related species can show high divergence in the number of MHC loci and some non-passerines may have relatively high number of MHC loci, probably as a result of recent duplications.

There was much stronger signature of diversifying selection at the MHC class II than class I in the White-tailed Eagle. Seven MHC class II amino acid residues (in contrast to only two class I residues) showed evidence for pervasive diversifying selection, and the *dN*/*dS* ratio estimates were higher for MHC class II sequences, when calculated either for all residues or putative PBRs. Stronger signature of diversifying selection at MHC class II was consistent with higher sequence polymorphism of class II genes, as assessed with the proportion of polymorphic sites, total number of mutations, and nucleotide diversity. This pattern is consistent with a recent global analysis of selection across the avian tree of life, showing stronger signature of diversifying selection at MHC class II versus class I in non-passerine birds [16]. Similar conclusions have been reached by few available direct comparisons of nucleotide substitution rates (*dN*/*dS* ratios) between MHC class I and class II genes in non-passerine birds. Higher *dN*/*dS* ratios at MHC class II versus class I have been reported for the Blue Petrel *Halobaena caerulea* (Procellariiformes) [20], six species from flamingo complex (Phoenicopteriformes) [21], *Centrocercus* and *Tympanuchus* grouse (Galliformes) [22], as well as in the Domestic Chicken *Gallus gallus* [68]. Genus *Falco* seems to constitute an exception to this rule, as both MHC classes in falcons were shown to accumulate synonymous and non-synonymous mutations at similar rates [28]. However, many falcon species show remarkably low MHC variation at both intra- and inter-specific level and, thus, it has been suggested that falcons may primarily depend on other immune mechanisms, e.g. powerful innate defences that override the need to trigger a costly adaptive response via MHC [28].

On the other hand, we cannot exclude that divergent MHC class II alleles of the White-tailed Eagle were adaptively retained through the bottleneck to preserve high capacity of extracellular pathogen recognition, while the loss of variation at class I could be primarily driven by stochastic processes, such as drift, rather than purifying selection. There is compelling empirical evidence for the role of genetic drift in causing rapid loss of MHC variation in bottlenecked populations of birds [69–71] and other vertebrates [72, 73]. This scenario is also consistent with the results of theoretical and meta-analysis approaches, showing that the effect of drift combined with selection acting prior to a bottleneck event should cause greater loss of MHC polymorphism than the loss of neutral genetic diversity [74, 75]. However, although we acknowledge that drift could have caused some loss of MHC diversity in our population of White-tailed Eagles, it is unlikely to solely explain marked differences in *dN*/*dS* ratios and sequence polymorphism between MHC class I and class II genes. Consequently, we conclude that these differences should, at least to some extent, reflect different selective pressures at the two MHC classes.

Although our results provided a clear support for higher allelic diversity and stronger diversifying selection at the MHC class II exon 2 than MHC class I exon 3, we have to acknowledge several methodological limitations of our study. First, our conclusions were based on the analysis of a single exon per each MHC class, while peptide-binding grooves of MHC molecules are formed by two domains (α_1_ and α_2_ at class I; α_1_ and β_1_ at class II), each coded by one exon. Although genotyping both domains per each class would produce much more robust results, it was methodologically not feasible for our study species, as there are no available primers for amplifications of α_1_ class I and class II domains in accipitrids and other closely related clades. Second, using a single pair of primers per class for PCR amplifications may have an excluding effect on some gene sequences or loci [2]. While the exclusion of certain loci could produce a significant bias in the analyses, we consider this bias rather unlikely in the case our study, as we recovered sequences from the highest number of MHC loci ever reported in accipitrids. Third, we had no information on the expression profiles of genotyped MHC loci and, thus, we could not exclude that our sequences originated from both classical (high variability and strong expression) and non-classical (low variability and reduced or tissue-specific expression) loci, which may have different evolutionary histories and be under different selective pressures [3, 76]. Although within our dataset we detected no non-functional alleles (having stop codons or frame-shift mutations), which could be characteristic for pseudogenes, a presence of non-classical sequences could not be excluded using our methodology. Taking all this into account, we recommend development of locus-specific primers for MHC class I and class II amplifications and assessing tissue-specific expression of MHC alleles using complementary DNA (cDNA) as crucial next steps for a better understanding of MHC evolution in the White-tailed Eagle. Having said that, it must be acknowledged that sample collection for examination of tissue-specific expression profiles of the MHC is highly invasive and must require a careful consideration when applied to an endangered species, such as the White-tailed Eagle.

Finally, phylogenetic clustering of White-tailed Eagle alleles with sequences recovered from other accipitrid species produced contrasting patterns at MHC class I and class II. In general, both MHC class I and class II sequences of White-tailed Eagle clustered together with sequences of the congeneric species, the Bald Eagle. However, all *Haliaeetus* class I sequences formed a single cluster, while class II sequences formed three distinct clusters separated with the sequences of other accipitrids. Higher similarity of sequences between rather than within species (trans-specific similarity) is a common characteristic of MHC genes and it is usually explained with trans-species polymorphism (TSP), which arises from the passage of alleles from ancestral species to descendant species through incomplete lineage sorting [13]. While neutral (transient) TSP maintains ancestral allelic variants in both descendant species for a short period of time after speciation (according to neutral expectation) and gradually disappears, strong balancing selection can maintain orthologous MHC lineages for much longer evolutionary times (so called balanced TSP) [13]. In fact, there are cases of MHC allele sharing between avian genera that are estimated to have diverged 20–30 mln years ago and, within Accipitridae, identical MHC alleles were recovered from *Aegypius* and *Gyps* genera, which diverged 12.7 mln year ago [16] (divergence time estimates according to Jetz et al. [77]). Although trans-specific similarity can be also explained with convergent evolution resulting from adaptation to similar selective pressures, molecular studies indicate that balanced TSP is a predominant mechanism responsible for trans-specific allele clustering at the avian MHC [78]. Considering stronger signature of diversifying selection at MHC class II versus class I in the White-tailed Eagle, we find it likely that trans-specific similarity of class II sequences in our study may also reflect balanced TSP rather than convergence.

## Conclusions

Our study shows that recently bottlenecked Central European population of White-tailed Eagles has retained significant levels of MHC diversity, which reinforces high conservation value of local White-tailed Eagle populations, as previously postulated by Hailer et al. [34]. At the same time, our study is the first to demonstrate contrasting patterns of allelic diversity and selection at MHC class I and class II in an accipitrid species, supporting the hypothesis that different mechanisms can shape evolutionary trajectories of MHC class I and class II genes.

## Additional file

Additional file 1: White-tailed Eagle MHC seqences. (FASTA 9 kb)

## Abbreviations

DDT: Dichlorodiphenyltrichloroethane
dN: Nonsynonymous substitution rate per nonsynonymous site
dS: Synonymous substitution rate per synonymous site
FEL: Fixed Effects Likelihood
FUBAR: Fast Unconstrained Bayesian AppRoximation
GARD: Genetic Algorithm for Recombination Detection
GTR: General time-reversible
MCMC: Markov Chain Monte Carlo
MEME: Mixed Effect Model of Evolution
MHC: Major Histocompatibility Complex
NNI: Nearest-neighbour interchanges
PBR: Peptide-binding residue
PCB: Polychlorinated biphenyl
PCR: Polymerase chain reaction
SPR: Subtree-pruning-regrafting
